# Supplementary material for: Comparison of Optical and Electrical Sensor Characteristics for Efficient Analysis of Attachment and Detachment of Aptamer
Source: Biosensors (Basel). 2022 Nov 7;12(11):979. doi: 10.3390/bios12110979 (PMC9688426; doi:10.3390/bios12110979)
Supplement: Supplementary file 1 [file biosensors-12-00979-s001.zip › biosensors-1903487-supplementary.pdf]

biosensors

MDPI

Article

Comparison of Optical and Electrical Sensor Characteristics for Efficient Analysis of Attachment and Detachment of Aptamer

Yejin Park [1,†], Thinh Viet Dang [2,†], Uiseok Jeong [3], Moon Il Kim [2,*] and Jinsik Kim [1,*]

1 Department of Biomedical Engineering, College of Life Science and Biotechnology, Dongguk University, Seoul 04620, Korea
2 Department of BioNano Technology, Gachon University, Seongnam 13120, Gyeonggi, Korea
3 SKhynix, Gyeongchung-daero 2091, Bubal-eup, Incheon-si 17336, Gyeonggi-do, Korea
* Correspondence: moonil@gachon.ac.kr (M.I.K.); lookup2@dongguk.edu (J.K.)
† These authors contributed equally to this work.

Citation: Park, Y.; Dang, T.V.; Jeong, U.; Kim, M.I.; Kim, J. Comparison of Optical and Electrical Sensor Characteristics for Efficient Analysis of Attachment and Detachment of Aptamer *Biosensors* **2022**, *12*, 979. https://doi.org/10.3390/bios12110979

Received: 22 August 2022
Accepted: 2 November 2022
Published: 8 November 2022

Publisher's Note: MDPI stays neutral with regard to jurisdictional claims in published maps and institutional affiliations.

Copyright: © 2022 by the authors. Licensee MDPI, Basel, Switzerland. This article is an open access article distributed under the terms and conditions of the Creative Commons Attribution (CC BY) license (https://creativecommons.org/licenses/by/4.0/).

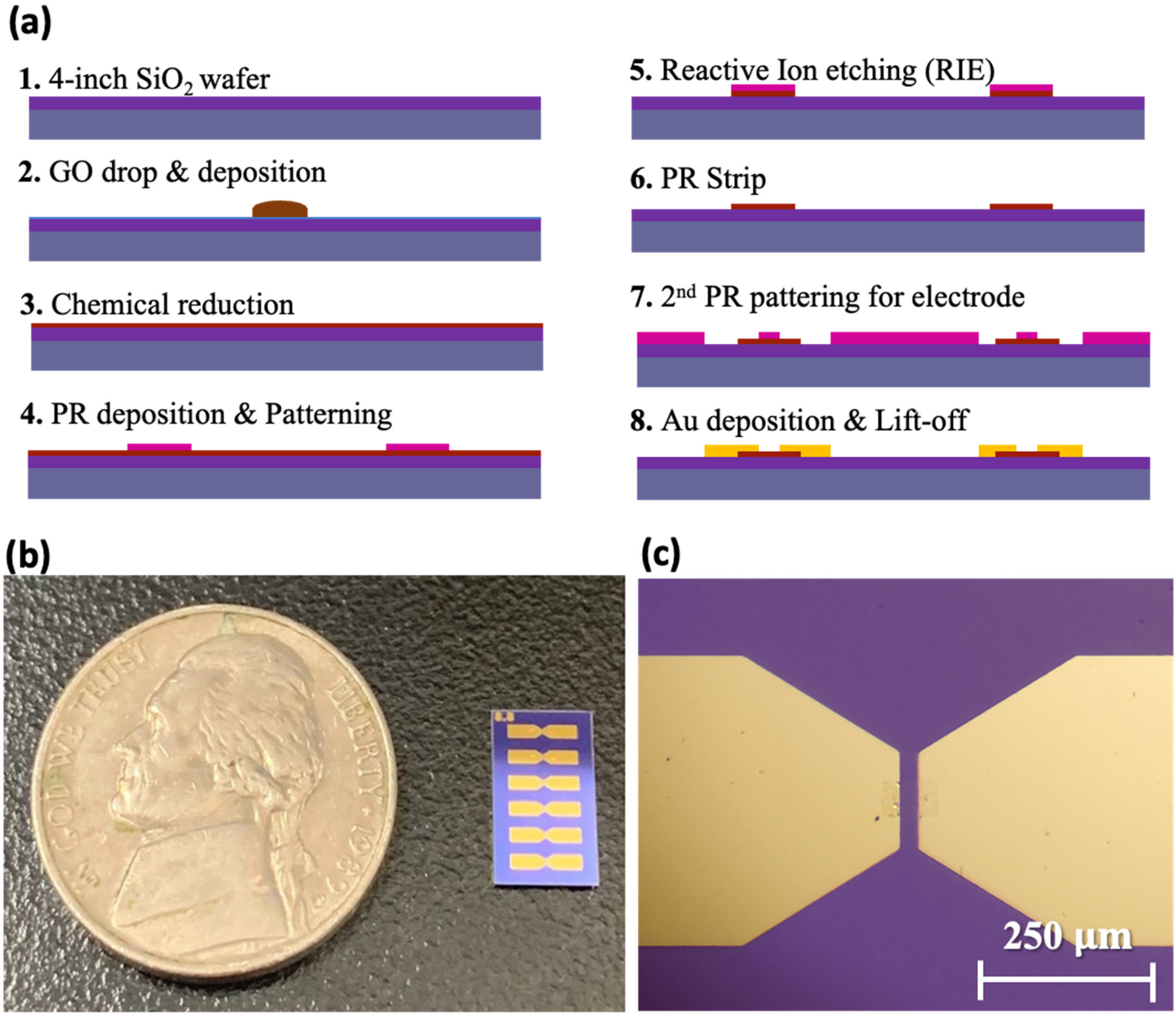

Figure S1. Fabrication scheme and actual image of rGO biosensor. Figure S1-(a) shows the fabrication process of rGO biosensor. PR patterning in the figure S1-(a) was perform using a photolithography method. The rGO biosensor has six sensing zones in an array, and the whole device is a rectangle with 6mm width and 10mm length, as shown in (b) and (c).

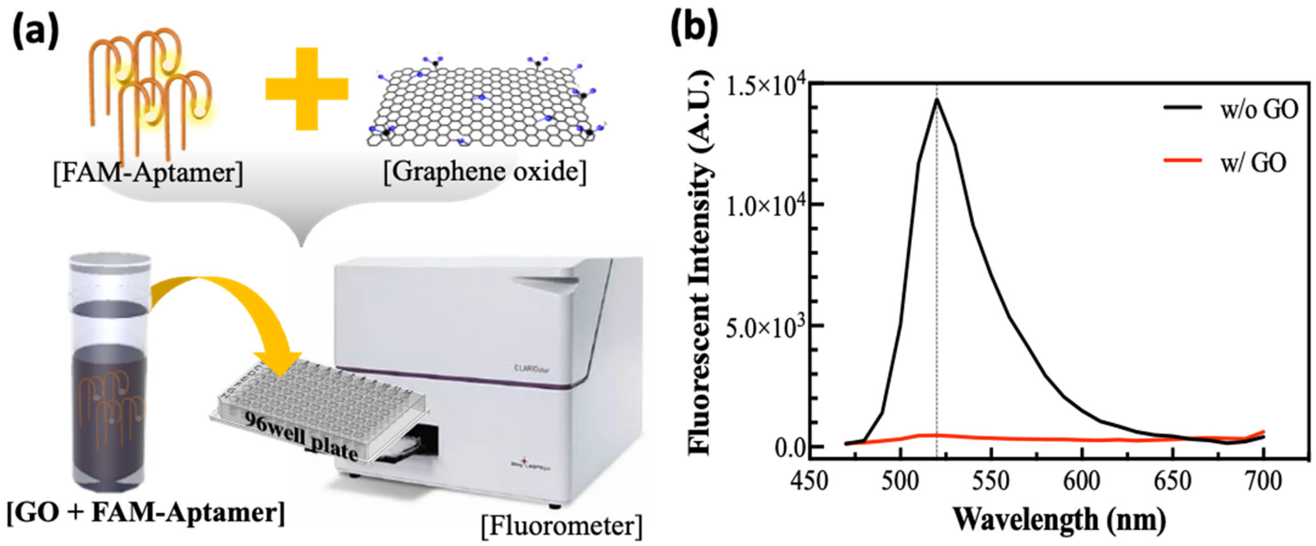

Figure S2. Simple reaction mechanism of optical measurement and fluorescence Intensity of TBA aptamers with(red) and without(black) GO.Fluorescein phosphoramidite(FAM) has an emission band of 517 nm. It is verified that the fluorescence intensity of FAM-TBA without GO has a peak value at the 520nm of wavelength, while the fluorescence of FAM-TBA was quenched afte GO reacts with FAM-TBA.

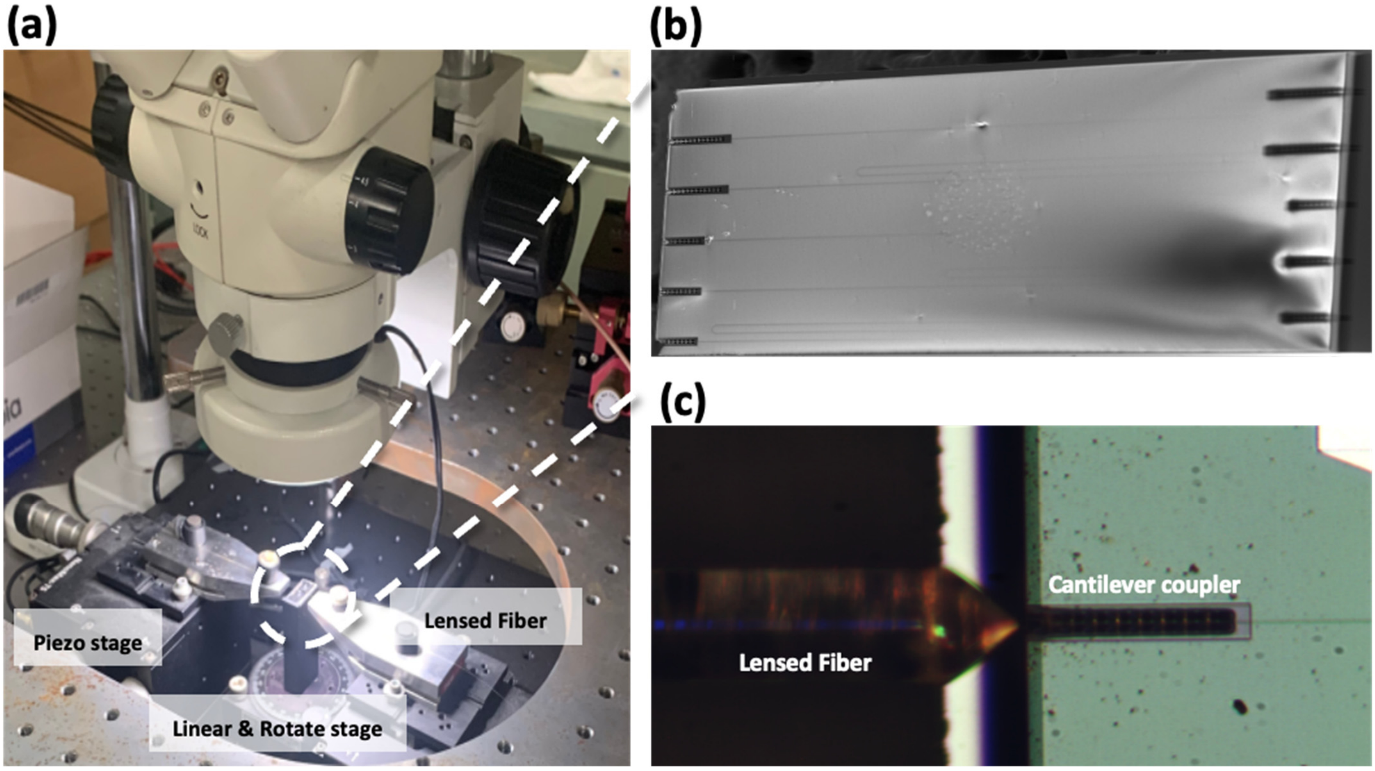

Figure S3. The experimental photograph of mechanical measurement. (a) is an overall view of the experiment. (b) is the cantilever arrangement of the device and (c) is the structure of cantilever coupler and lensed fiber used in the experiment.

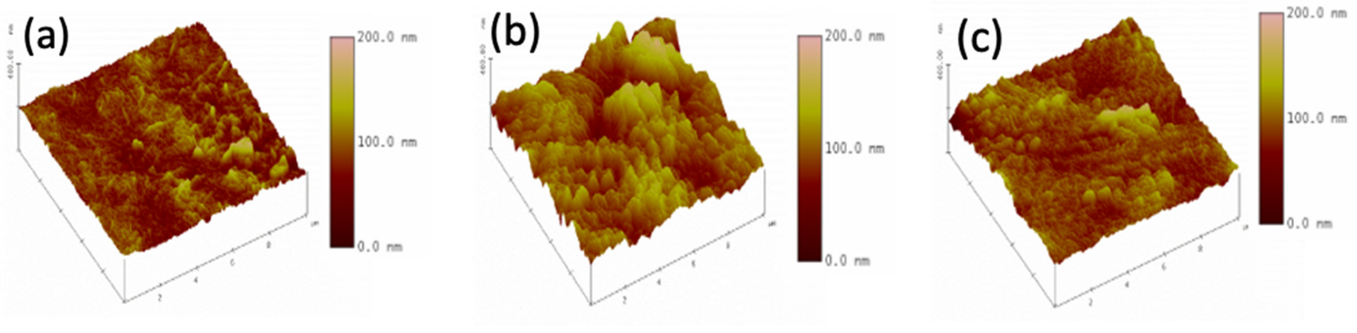

Figure S4. The visualization of AFM data according to the reaction step among GO, Aptamer and thrombin. (a) is surface of bare-GO and (b) is surface of GO after thrombin binding aptamer reaction. (c) shows surface after thrombin reaction on the surface of (b). The absorption and desorption reaction among graphene oxide, aptamer and thrombin were verified by AFM (atomic force microscope). The root mean square roughness (Rq) of the surface was measured and analyzed. The Rq is based on the height which is the difference between upper line and baseline when it raised from the higher valley than baseline to a lower valley than baseline within the specified interval, L. The value of Rq could be calculated by Rq=$\sqrt{\frac{1}{L}\int_0^L Y^2(x)\,dx}$. The acquired value as follow the equation shown in table S1.

Table S1. The Rq-value of figure S4.

	Reaction step (Target)	Rq
S4-(a)	Bare-GO	9.384 nm
S4-(b)	Thrombin binding aptamer	30.927 nm
S5-(c)	Thrombin	13.446 nm

To compare Figure S4-(a) and (b), the Rq was increase after the reaction of thrombin binding aptamer(TBA) with bare graphene oxide. After that, the Rq was decrease when the thrombin reacts with graphene oxide surface with TBA absorption.

Equation S1

$$\boldsymbol{electrical\ resistance\ change\ ratio}(\%) = \left(\frac{\boldsymbol{R_{Af} - R_{Bf}}}{\boldsymbol{R_{Bf}}}\right) * \mathbf{100} \quad \textbf{Equation S1}$$

The electrical resistance change rate(dR/R_0) could be calculated by Equation S1. R_{Af} and R_{Bf} refer to electrical resistance values after the reaction and before the reaction, respectively. In verifying step of aptamer, R_{Bf} was initial electrical resistance of bare device.
